# Lipidation Approaches Potentiate Adjuvant-Pulsed Immune Surveillance: A Design Rationale for Cancer Nanovaccine

**DOI:** 10.3389/fbioe.2020.00787

**Published:** 2020-07-28

**Authors:** Junqing Wang, Harshal Zope, Mohammad Ariful Islam, Jamie Rice, Sage Dodman, Kevin Lipert, Yunhan Chen, Bruce R. Zetter, Jinjun Shi

**Affiliations:** ^1^Center for Nanomedicine and Department of Anesthesiology, Brigham and Women’s Hospital, Harvard Medical School, Boston, MA, United States; ^2^School of Pharmaceutical Sciences (Shenzhen), Sun Yat-sen University, Guangzhou, China; ^3^Immuno-Oncology Group, Immunomic Therapeutics, Inc., Rockville, MD, United States; ^4^Vascular Biology Program, Boston Children’s Hospital, Harvard Medical School, Boston, MA, United States; ^5^Silicon Therapeutics, Boston, MA, United States

**Keywords:** lipidation, nanovaccine, adjuvant, peptide antigen, Toll-like receptors, cancer

## Abstract

Adjuvant-pulsed peptide vaccines hold great promise for the prevention and treatment of different diseases including cancer. However, it has been difficult to maximize vaccine efficacy due to numerous obstacles including the unfavorable tolerability profile of adjuvants, instability of peptide antigens, limited cellular uptake, and fast diffusion from the injection site, as well as systemic adverse effects. Here we describe a robust lipidation approach for effective nanoparticle co-delivery of low-molecular weight immunomodulators (TLR7/8 agonists) and peptides (SIINFEKL) with a potent *in vivo* prophylactic effect. The lipidation approaches (C_16_-R848 and C_16_-SIINFEKL) increased their hydrophobicity that is intended not only to improve drug encapsulation efficiency but also to facilitate the membrane association, intracellular trafficking, and subcellular localization. The polymer–lipid hybrid nanoparticles (PLNs) are designed to sustain antigen/adjuvant levels with less systemic exposure. Our results demonstrated that a lipidated nanovaccine can induce effective immunity by enhancing the expansion and activation of antigen-specific CD8^+^ T cells. This adaptive immune response led to substantial tumor suppression with improved overall survival in a prophylactic setting. Our new methodology enhances the potential of nanovaccines for anti-tumor therapy.

## Introduction

The use of peptide epitope-based cancer vaccines to activate tumor-associated antigen (TAA)-specific T cell responses is an attractive option for generating long-term anti-cancer immune protection because of the ease of synthesis, tolerability, and low risk of adverse effects (Nabel, 2013; Skwarczynski and Toth, 2016; Kumai et al., 2017). TAA-based subunit vaccines are known to be poorly immunogenic, however, and require potent adjuvants to augment antigen-presenting cell (APC) activation and TAA presentation (Perrie et al., 2008; Coffman et al., 2010; Reed et al., 2013). Among various cancer vaccine adjuvants, TLR7/8 agonists are of particular interest because of their strong activation of APCs (Napolitani et al., 2005), initiation of cross-priming, promotion of CD4^+^/8^+^ T and natural killer (NK) cell activation (Kastenmuller et al., 2011), ability to limit the immunosuppressive function of regulatory T (Treg) cells, and association with an inflammatory tumor microenvironment via induction of cytokines and chemokines (Peng et al., 2005; Vasilakos and Tomai, 2013).

The imidazoquinoline compounds imiquimod and resiquimod (R848) are novel TLR7/8 agonists that demonstrate the potential for potent antiviral and antitumor activity when used as adjuvants (Junt and Barchet, 2015; Hu et al., 2017). The physicochemical properties of imidazoquinolines lead to rapid distribution from the site of injection which results in systemic exposure and cytokine-induced immune activation accompanied by adverse influenza-like symptoms (Vasilakos and Tomai, 2013). Nanoparticle (NP)-mediated synchronous delivery of adjuvant and antigen has emerged as a promising strategy, which can accommodate engineering approaches to promote APC uptake and reduce systemic side effects. Different NP platforms such as micelles (Black et al., 2012), liposomes (Gadd et al., 2018), and polymeric NPs (Ilyinskii et al., 2014; Kim et al., 2018) have been established in recent studies for TLR7/8 agonist-based cancer vaccine. These agonists were either physically encapsulated in the NPs or conjugated with poly(lactic acid) (PLA) to enhance the loading efficiency (Kim et al., 2018; Nuhn et al., 2018; Rodell et al., 2018; Thauvin et al., 2019). The physical encapsulation of imidazoquinoline compounds is often restricted to moderate encapsulation efficiency and fast burst release of payloads, whereas the polymer-based conjugation approaches are challenged by low carrying capacity, heterogeneity of polymer molecular weight, and the variable reproducibility (Dane and Irvine, 2015). Although the earlier studies (Ilyinskii et al., 2014; Alexis et al., 2016) demonstrated an effective local immune activation and an excellent safety profile (that attenuates the level of serum inflammatory cytokines for 50- to 200-folds compared with free R848 administration), up until now the prophylactic use of a TLR7/8 agonist-based nanovaccine for cancer prevention in animal models has not been rigorously evaluated. To achieve sustained cancer immune surveillance from a vaccine, the adjuvant strategy needs to consider key factors including the physicochemical properties of imidazoquinolines and peptides, as well as their ability to be formulated with NP encapsulation.

Lipidation is an important modification strategy for bio-active molecules, which has shown exceptional promise in pharmaceutical applications. For example, lipidation can address cytokine storm-like effects from post-subcutaneous injection of TLR agonists (Smirnov et al., 2011). The addition of an alkyl chain lipid moiety to TLR agonist can effectively improve the pharmacokinetic profile via slow dissemination from the site of application (Smirnov et al., 2011). Another key advantage of lipidation is that it enables hitchhiking of molecular vaccines on albumins (through conjugating lipophilic albumin-binding tail on antigen and adjuvant) and transports these molecules to lymph nodes (LNs), leading to dramatic increases in T-cell priming (Liu et al., 2014; Moynihan et al., 2018). Lipidation of the cationic dendrimers with alkyl chains affords lipid-like properties, promoting hydrophobic aggregation with siRNA/mRNA sequences, resulting in formation of more stable nano-formulation by keeping nucleic acids in inner core of NPs (Khan et al., 2014; Whitehead et al., 2014). Such condensed assembly methodology provides a gene delivery *in vivo* with long circulating half-lives and effective accumulation at target site (Kowalski et al., 2019).

**SCHEME 1:**
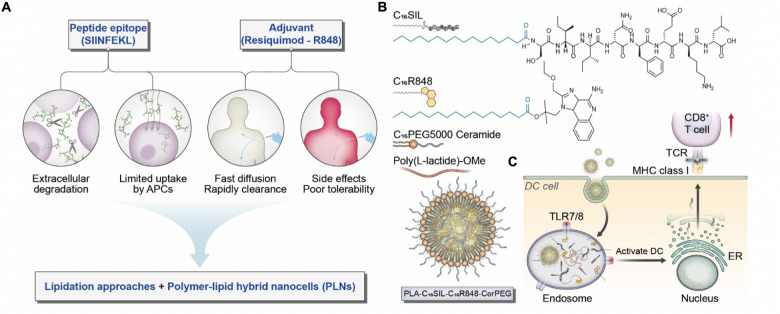
**(A)** Considerations and proposed formulation design for overcoming biological limitations of TLR7/8 agonists and peptide epitopes. **(B)** Schematic illustration of chemical structures for C_16_SIL [N-terminal palmitoylation of SIINFEKL (C16:0)] and C_16_R848 (esterification of palmitic acid with R848 hydroxyl group). The final PLNs (PLA-C_16_SIL-C_16_R848-CerPEG) were assembled via PLA assisted co-encapsulation of C_16_R848 and C_16_SIL followed by PEGylated ceramide stabilization. **(C)** Desired TLR7 activation and MHC I mediated antigen presentation elicited by PLN vaccine.

In this report, we investigated how lipidation could be exploited to optimize co-formulation and to regulate the pharmacokinetic profiles of peptide antigen and small molecular adjuvant to provide an enhanced vaccine response (Scheme 1A). Given the ability of lipid motifs to facilitate condensed particle formation of water-soluble molecules through hydrophobic aggregation, we have developed a novel lipidation approach for delivering a chemical analog of R848 plus the ovalbumin-derived peptide 257-264 (SIINFEKL) (Scheme 1B). To address the common formulation challenges including the post-entrapment leakage and rapid release of low molecular weight (LMW) payloads, we capitalized on core-shell polymer–lipid hybrid nanoparticles (PLNs) to incorporate LMW lipophilic molecules. The study here demonstrated a rationale design based on chemical engineering and vaccine formulation science to achieve minimal systemic exposure, consistent APC uptake, and prolonged immune surveillance, leading to an effective prophylactic anti-cancer vaccine.

## Materials and Methods

### Materials

Resiquimod (R848), anhydrous dichloromethane (DCM), anhydrous N,N-dimethylformamide (DMF), palmitic acid, N,N’-dicyclohexylcarbodiimide (DCC), 4-dimethylaminopyridine (DMAP), hydrochloric acid (HCl), magnesium sulfate, N-methylmorpholine (NMM), N,N’-diisopropylcarbodiimide (DIC), trifluoroacetic acid (TFA), triisopropylsilane (TIS), diethyl ether, palmitic acid (C16-COOH), piperidine, hexane, dimethyl sulfoxide (DMSO), ammonium hydroxide (NH4OH), β-mercaptoethanol, methyl-B-cyclodextrin (MBCD), and chlorpromazine were purchased from Sigma-Aldrich. FMOC amino acid conjugates (those of serine, isoleucine, asparagine, phenylalanine, glutamic acid, lysine, and leucine) were from EMD NovaBioChem, a part of EMD Millipore. 1-[Bis(dimethyla mino)methylene]-1H-1,2,3-triazolo[4,5-b]pyridinium 3-oxid he xafluorophosphate (HATU) was from Chempep (Wellington, FL, United States). 1-Hydroxy-7-azabenzotriazole (HOAt) was from Advanced Chemtech (Louisville, KY, United States). Acetonitrile (ACN) for HPLC was from VWR (Radnor, PA, United States). FMOC-PEG12-propionic acid was from AAPTec (Louisville, KY, United States). The ester-terminated poly(lactide) (PLA) polymer with viscosity of 0.55–0.75 dL/g was purchased from Lactel Absorbable Polymers (Birmingham, AL, United States). Polyethylene glycol (PEG) polymer, N-palmitoyl-sphingosine-1-succinyl-methoxy(polyethylene glycol)5000 (ceramide-PEG5k), was from Avanti Polar Lipids (Alabaster, AL, United States). EG7-OVA cell line was from ATCC (Manassas, VA, United States).

### C_16_R848 Synthesis

R848 (50 mg), DCM (3 mL), and DMF (1 mL) were added to a 100 mL round bottomed flask. The solution was stirred at 1600 rpm under nitrogen gas followed by the addition of palmitic acid and DCC, with 100 mg of each. The reaction mixture was stirred for 10 min and 21 mg of DMAP was added. The reaction mixture was then stirred overnight under nitrogen gas. The reaction mixture was vacuum filtered to remove precipitates formed during the reaction. The reaction mixture was then washed six times with water to remove hydrophilic reactants and byproducts. After the final wash, the remaining water was removed by magnesium sulfate. The wax-like crude product was obtained by rotary evaporation. C_16_R848 was then purified on a Gilson GX-271 HPLC (Gilson, Middleton, WI, United States) using a Vydac 214TP101522 22 × 250 mm C4 column (Grace, Columbia, MD, United States). Identity and purity of the compound was verified using MALDI-TOF (Bruker, Billerica, MA, United States).

### C_16_SIINFEKL Peptide Synthesis

The peptide derivatives were synthesized using a solid phase synthesis method. The scale of the synthesis is 0.1 mmol for C_16_SIINFEKL. The synthesis was done on a Tribute-UV automatic protein synthesizer (Protein Technologies, Tuscon, AZ, United States), using preloaded Low Load Wang resins with polystyrene support (EMD Millipore NovaBioChem). The resin was housed in a 10 mL batch synthesis fritted reaction vessel. The amino acids were deprotected in a solution of 20% piperidine in DMF, with each deprotection cycle lasting 2:30 min. The cycle was repeated until the UV feedback from the Fmoc cation detector of the synthesizer comes back as 3% or lower of the original UV signal before the first deprotection cycle. The residues were then activated and coupled in a DMF solution with a molar ratio of 1:1:2 for amino acids, HATU, and NMM, respectively. Palmitic acid (C_16_–COOH) was coupled overnight to amino acid residues in a mixture of DIC and HOAt in DMF solvent. After solid phase-assisted polymerization, the peptide derivatives were cleaved using a mixture of 92.5% TFA, 5% TIS, and 2.5% water (v/v/v) for 2 h. The derivatives were then precipitated in a diethyl ether and hexane mixture in a ratio of 10:1. The precipitation mixture was centrifuged; the ether and hexane supernatant were removed. The precipitate was then reconstituted in an acetic acid or ACN solution. The solution was frozen and then lyophilized. C_16_SIINFEKL was purified on a Gilson GX-271 HPLC on a TMS-250 10 × 100 mm C1 column (Tosoh, Tokyo, Japan). Identity and purity of the compounds was verified using MALDI-TOF.

### PLNs Preparation

All nanoparticles were prepared through nanoprecipitation procedure, where the aqueous to organic phase volume ratio (A/O) did not exceed 1:20. PLA was dissolved in DMSO at 10 mg/mL. Ceramide-PEG5k was dissolved in water at 10 mg/mL. C_16_R848 was dissolved in DMF at 5 mg/mL. C_16_SIINFEKL was dissolved in 2 Dr vials at 5 mg/mL in DMSO. The water phase was prepared by adding 10 mL HyClone water to a 20 mL vial containing a stir bar. It was then heated on a hot plate (VWR or Chemglass) to 80°C for 5 min while stirring at 800 rpm; 200 uL of ceramide-PEG solution was added, and the mixture was then heated at 80°C for another 5 min before nanoprecipitation. For the organic phase, 175 uL of PLA was used for drug-loaded PLNs, while 200 uL was used for empty control PLNs. Then, depending on the intended composition, 50 uL of C_16_R848 solution and/or 50 uL of C_16_SIINFEKL (for NP-C_16_SIL-C_16_R848) were added to organic phase. These stock solutions were sonicated in a water bath sonicator to assist dissolution. Finally, DMSO was added to the organic phase until final volume was 487.5 uL. The fully prepared organic phase was then sonicated and nanoprecipitation was performed with the organic phase being added to the water phase while the tip was submerged. The solution was stirred for 1.5 min on the hotplate before it was transferred to a stir plate and stirred at 1200 rpm for 1 h. The particle solution was then purified with a 40 μm cell strainer. The organic solvents were removed, and the NPs were concentrated via centrifugation with two water washes using 15 mL Amicon Ultra Filters with 100 K cutoff.

### HEK-Blue mTLR7 Assay

HEK293 cells expressing human TLR7 or TLR8 with an NF-κB-inducible responsive SEAP reporter gene were obtained from InvivoGen (San Diego, CA, United States). Cells were cultured in DMEM with 10% FBS and antibiotics. Cells were plated at 96-well plates and stimulated for 24 h. Supernatants were harvested and monitored by NF-κB/SEAP activation using HEK-Blue^TM^ Detection Kit (InvivoGen) according to the manufacturer’s instructions.

### DC Antigen Presentation Experiment

Day 1 – *Plating Cells*. The media composed of RPMI, 1% penicillin-streptomycin, 10% FBS (HyClone), and 0.05 mM of β-mercaptoethanol. The following directions assume that the dendritic cells (DC2.4) were cultured in a T75 flask. Cells were resuspended in 1 mL of fresh media and counted; 1 mL of this suspension was added to each well in a 24-well plate. The plate was then incubated overnight. Optional Day 2 – *Inhibitor Addition for Endocytosis Experiment*. About 500 uL of media was removed and varying amounts of inhibitors were added. For MBCD, 1.5 mg was added per well, and for chlorpromazine, 3.25 ug was added per well. Day 2/3 – *Treatment Additions*. A 1× PBS solution was made for each nanoparticle suspension and free SIINFEKL so that the concentration was 1 ug of encapsulated or free SIINFEKL per 100 uL. Day 3/4 – *FACS Processing*. FACS buffer was made with 1× PBS and 5% FBS. A solution with 2 uL (1 ug) of CD16/32 antibody per 50 uL of FACS buffer was prepared; 50 uL of the previously prepared CD16/32 solution was added to each Eppendorf tube. They were mixed vigorously with a pipette and then incubated on ice for 10 min; 3 uL of PE/Cy7 anti-mouse H2Kb-SIINFEKL antibody was added to each sample. After centrifugation, 200 uL of 0.2% PFA solution was added to each sample, including cells only, and they were mixed vigorously without creating bubbles; 200 uL of each sample was transferred to a clear, round bottomed 96-well plate. Once completed, the plate was protected with aluminum foil and ran on flow cytometer, using the NIR-B channel for detection.

### *In vivo* Experiments

#### Vaccination Procedure

Each group of five black (MOUSE TYPE) female mice underwent three rounds of vaccinations. There were three groups—saline, alum control (NPC), and NP-C_16_SIL-C_16_R848, and each injection consisted of 100 or 110 uL depending on the group. The suspensions of the experimental particles were rendered into those of PBS 1× by the addition of PBS 10× that is 10% the volume of the particle solutions. Then, each syringe was loaded with 110 uL of this suspension. The alum control, NPC, was created by mixing C_16_-R848 and C_16_-SIINFEKL dissolved stock solutions with Imject^®^ Alum reagent and PBS 1×. The stocks were in a solution of 5 mg/mL of DMF and DMSO, respectively, and the amount of drugs per alum injection matched the amount of drugs per injection in the experimental NP-C_16_SIL-C_16_R848. A volume equaling six such 100 uL injection stocks was made for injecting five mice per vaccination round. Each mouse in the PBS group was injected with only 100 uL of PBS 1×. Half of the injection went into the footpad while the other half was intraperitoneal. The vaccinations were repeated two times, with the second vaccination given 2 weeks after the first and the third given 1 week after the second. The subsequent tumor inoculation and measurement procedures are demonstrated in Supplementary Section.

## Results and Discussion

### Lipidation Design Strategy

Palmitic acid (C16:0) is an endogenous fatty acid that plays an important role in posttranslational modification of cytosolic proteins via enzymatic palmitoylation (Linder and Deschenes, 2007; Sobocinska et al., 2017). The hydrophobic attachment of palmitoyl chains to proteins is a key step that facilitates maturational processing, trafficking, and membrane anchoring in cellular compartments (Scheme 1C) (Linder and Deschenes, 2007). Recent evidence indicates that both biological palmitoylation and synthetic lipidation can critically influence immune responses (Park et al., 2004; Smirnov et al., 2011; Black et al., 2012; Lochner et al., 2015; Foster et al., 2018). We therefore speculate that covalent attachment of palmitic acid on SIINFEKL and R848 would improve vaccine function by improving their local bioavailability, reducing systemic toxicity and enabling effective co-formulation with PLNs.

### Formation and Characterization of PLN Vaccine

**FIGURE 1:**
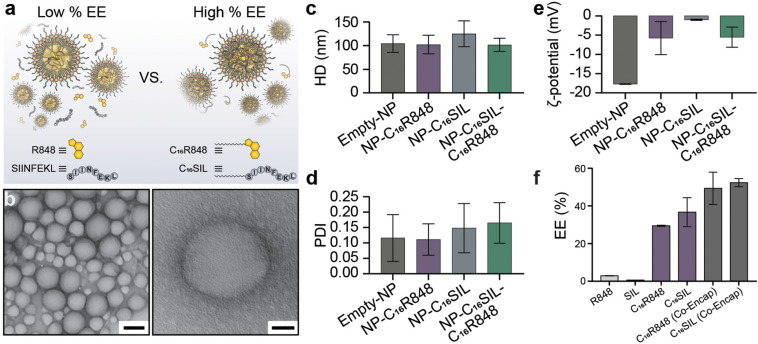
Physicochemical properties of Core-Shell PLNs. **(a)** The comparison of EE between with and without lipidation of R848 and SIL. **(b)** Representative TEM image of lipidated conjugates formulated with PLNs (Scale bar: left, 100 nm; right, 20 nm). **(c)** The mean hydrodynamic diameter (HD), **(d)** polydispersity index (PDI), and **(e)** zeta potential (ζ) of PLNs. **(f)** Optimum mean EE for mono-encapsulation of R848, SIL, C_16_R848, C_16_SIL, and co-encapsulation of C_16_R848 and C_16_SIL.

The lipidated TLR7/8 agonist (C_16_R848) and epitope (C_16_SIINFEKL) were synthesized via Steglich esterification and solid phase-assisted polymerization, respectively. The final structure of products was confirmed by high performance liquid chromatography/mass spectrometry (HPLC/MS, as illustrated in Supplementary Figures S1–S6). PLNs were prepared by the nanoprecipitation method and formulated with C_16_R848 and/or C_16_SIL to result in four different groups: (i) empty-NP, (ii) NP-C_16_R848, (iii) NP-C_16_SIL, and (iv4) NP-C_16_SIL-C_16_R848. The PLN formation was optimized via temperature-assisted nanoprecipitation. We found that the elevated temperature of aqueous phase could effectively reduce viscosity during addition of the organic portion, which resulted in desired encapsulation efficacy (EE) and spherical form under transmission electron microscopy (TEM) (Supplementary Figure S7). The final obtained PLNs formulated with both lipidated conjugates exhibited monodispersed colloidal features and a polyethylene glycol (PEG) corona can be observed from high-resolution transmission electron microscopy (HR-TEM) (Figure 1b). Interestingly, the encapsulation of lipidated conjugates has little or no impact on the hydrodynamic diameter (HD) but slightly increased the polydispersity indices (PDI) (Figures 1c,d). Due to the neutral charge and lipophilicity of C_16_R848 and C_16_SIL, the core-shell structure with the outer layer stabilized by a C_16_-Ceramide-PEG5000 coating gave rise to NP-C_16_SIL-C_16_R848 with a slightly negative surface potential (-5.5 mV) compared with empty-NP (-17.7 mV) (Figure 1e). The C_16_R848 and C_16_SIL co-formulated with polylactide (PLA) polymer were intended to promote EE. The PLN co-formulation without lipidation exhibited extremely low, which was attributable to the aqueous solubility of SIINFEKL and small soluble aggregates of R848 (Figure 1a). As shown in Figure 1f, lipidated conjugates demonstrated 10- and 35-folds improvement in EE (C_16_R848, 29.4% and C_16_SIL, 36.6%) relative to their unmodified counterparts, and additional EE gains were achieved after co-encapsulation of C_16_R848 (49.3%) and C_16_SIL (52.4%). Taken together, these findings suggest that NP-C_16_SIL-C_16_R848 is capable of encapsulating both antigen and adjuvant in a manner that is suited for subsequent *in vitro* study.

### *In vitro* Immunomodulatory Activity of PLN Vaccine

**FIGURE 2:**
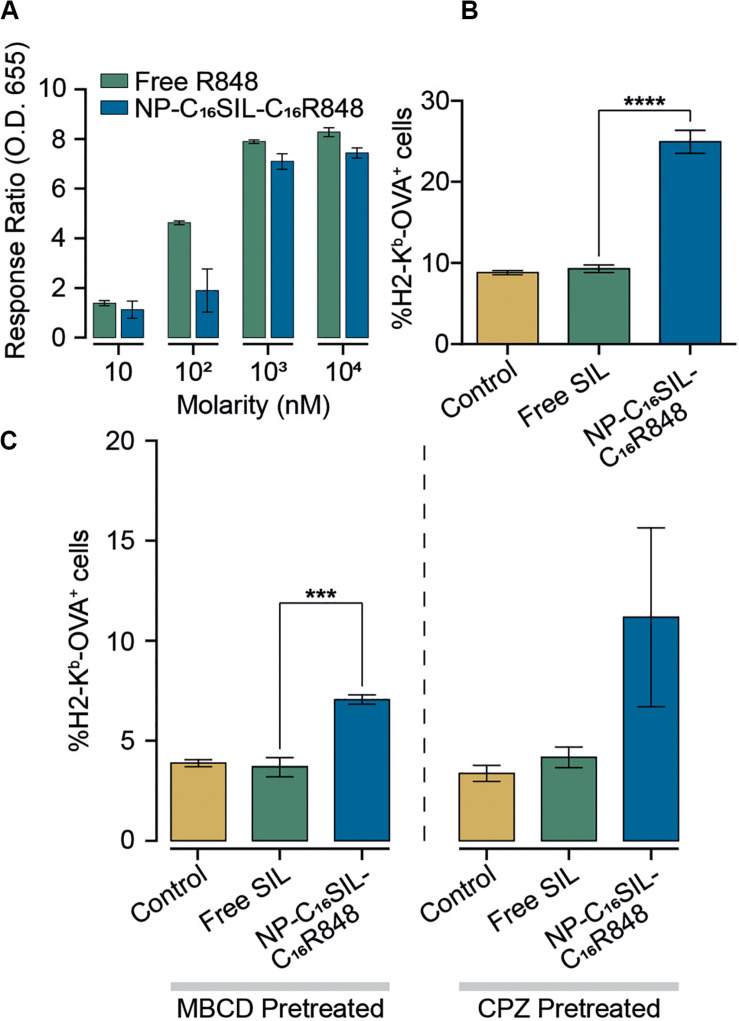
Enhanced immunomodulatory activity of PLNs *in vitro*. **(A)** Detection of TLR7/8 agonist activity when stimulated with increased concentrations of free R848 and co-encapsulated NP (NP-C_16_SIL-C_16_R848). **(B)** The mean percentage of MHC I molecules presenting OVA-derived peptide SIL on DC2.4 surface. **(C)** DC2.4 groups were pretreated with two different cellular uptake inhibitors (MβCD and CPZ) and incubated overnight before treatment with controls and NPs. The mean percentage of SIL display was analyzed by fluorescence-activated cell sorting (FACS).

To assess the *in vitro* efficacy of PLNs that co-deliver C_16_R848 and C_16_SIL, TLR7/8 agonist activity was measured in a HEK293T cell-based IRF reporter assay. In a direct comparison of free R848 and co-formulation form (NP-C_16_SIL-C_16_R848) at higher concentration (10^3^–10^4^ nM) demonstrated a similar *in vitro* potency to free R848 (Figure 2A), while a low concentration (10–10^2^ nM) of C_16_R848 (NP-C_16_SIL-C_16_R848) had no significant effect on activity, implying that relatively high concentration loading of C_16_R848 by PLNs could be essential for efficient TLR7/8 activation.

A critical process driving CD8^+^ primary T cell responses to vaccines *in vivo* is antigen cross-presentation through the formation of exogenously derived peptide with MHC complexes presented on the surface of dendritic cells (DCs). The ability of the PLNs to increase antigen cross-presentation was investigated in murine DC2.4 dendritic cells by fluorescent immunostaining (Figure 2B). Notably, groups treated with NP-C_16_SIL-C_16_R848 showed higher MHC-I mediated SIL antigen presentation on the cell surface compared to control and free SIL treatment groups. This suggests that DC2.4 cells upregulate peptide-MHC I complex presentation in response to NP-C_16_SIL-C_16_R848, consistent with the general concept that dendritic cell activation by TLR7/8 agonists is associated with increased antigen cross-presentation and downstream adaptive responses (Iwasaki and Medzhitov, 2004). Because endocytic pathways can influence nanoparticle-mediated delivery of peptide antigens to dendritic cells, we measured the MHC-I presentation of SIINFEKL while simultaneously blocking the class A scavenger receptor (SR-A), lipid raft or clathrin-dependent endocytosis. The results (Figure 2C) indicate that blocking lipid raft-mediated endocytosis with methyl-β-cyclodextrin (MβCD) dramatically inhibited the surface antigen presentation for all groups compared with non-treated controls (Figure 2C). However, the epitope expression level mediated by NP-C_16_SIL-C_16_R848 remained twofold higher than free SIL without R848. A similar result was observed when chlorpromazine (CPZ) is used to disrupt the SR-A-mediated uptake pathway (Figure 2C). Collectively, these results indicate that PLN-mediated co-delivery of C_16_R848 adjuvant and C_16_SIL antigen results in dendritic cell activation and a significant increase in antigen presentation at higher loading concentrations.

### *In vivo* Immunomodulatory Activity of PLN Vaccine

**FIGURE 3:**
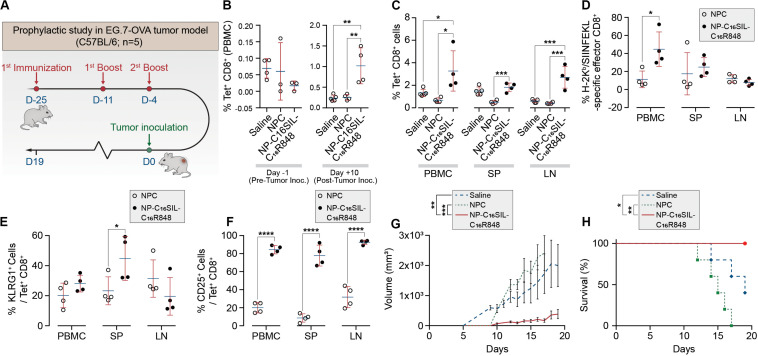
PLN vaccine prevents tumor growth in murine cancer models with a prophylactic setting. **(A)** Regimens of PLN vaccine tested in C57BL/6 mice. **(B)** Longitudinal study of percentages of SIL-positive CD8^+^ T cells among peripheral blood mononuclear cells (PBMCs) on day -1 before and day 10 after tumor inoculation. **(C)** Percentages of SIL-positive CD8^+^ T cells among PBMCs, spleens (SP) and lymph node (LN) on day 19. **(D)** Mean percentages of SIL-positive effector CD8^+^ T cells. **(E)** The frequency of KLRG1-positive subsets from SIL-positive CD8^+^ T cells. **(F**) Percentages of SIINFEKL-specific CD25^+^ Tregs on day 19. **(G)** The size of E.G7-OVA tumors was monitored over time. **(H)** Survival curve of immunized mice inoculated with E.G7-OVA tumor cells. Data are representative of two independent experiments. Experimental groups consisted of five mice. *P < 0.05, ***P < 0.001, and ****P < 0.0001 analyzed by one-way ANOVA **(B–F)** or two-way ANOVA **(G–H)** with Tukey’s multiple comparisons post-test.

Having demonstrated dendritic cell activation *in vitro*, we sought to explore the ability of NP-C_16_SIL-C_16_R848 to elicit an antigen-specific immune response *in vivo* (Figure 3A). Naïve C57BL/6 mice were immunized with a primary immunization followed by two booster injections with a fixed 100 μL/vaccine dose that included NPC [Alum/C_16_-SIL (10 μg)/C_16_-R848 (7 μg)], NP-C_16_SIL-C_16_R848 [C_16_SIL (10 μg)/C_16_R848 (7 μg)], or vehicle control. On day 3 following the third immunization, we quantified the frequency of SIL-specific CD8^+^ T cells by the SIL/H-2Kb tetramer (OVA-Tet-PE) staining. We did not detect a significant increase in Tet^+^ CD8^+^ T cells prior to the tumor inoculation in any of the treatment groups (Figure 3B, Day 1), indicating any expansion of Tet^+^ CD8^+^ T cells due to vaccination was modest in peripheral blood. To evaluate the immunogenic effects of the nanovaccine strategy in tumor xenograft model, C57BL/6 mice were then inoculated s.c. with E.G7-OVA thymic lymphoma cells. At 10- and 19-days post tumor inoculation, the NP-C_16_SIL-C_16_R848 vaccinated group exhibited a significant increase in the frequency of SIL-specific CD8^+^ T cells compared with vehicle control (*P* = 0.0021) and NPC groups (*P* = 0.0035) (Figure 3B, Day 10). On day 19 post tumor inoculation, there was a higher percentage of Tet^+^ CD8^+^ T cells in the secondary lymphoid tissues including the spleen (SP) and lymph node (LN) (Figure 3C); NP-C_16_SIL-C_16_R848 vaccination resulted in significantly higher frequencies of SIL-specific CD8^+^ T cells within the PBMC (*P* = 0.0114), SP (*P* = 0.0005), and LN compartments (*P* = 0.0003) compared to alum-adsorbed OVA R848 vaccine. Besides, effector CD8^+^ T cells were found to be differentially promoted in both PBMC and SP, but negligible difference was observed as compared with NPC groups (Figure 3D).

Effector CD8^+^ T cells are a key mediator of cell-mediated cytotoxic antitumor immunity (Melief, 2008), and a subset of these T Cells are critical for cancer immune surveillance. The pool of these memory precursor effector cells can be further divided into short-lived effector CD8^+^ T cells (SLECs) and memory precursors (MPs) (Kaech et al., 2002), and KLRG1 (Killer Cell Lectin Like Receptor G1) is a cell marker expressed by CD4^+^ and CD8^+^ T cells that exhibit a memory cell phenotype (Olson et al., 2013; Herndler-Brandstetter et al., 2018). Furthermore, these cells can differentiate into memory cells that are capable of mounting highly effective anti-tumor responses upon tumor rechallenge (Herndler-Brandstetter et al., 2018). To evaluate whether different vaccine strategies could induce this desired T cell phenotype, we assessed KLRG1 expression in antigen-experienced CD8^+^ T cells in PBMC, SP, and LN compartments. Notably, compared with NPC formulation groups on day 19, NP-C_16_SIL-C_16_R848 immunized groups exhibited higher levels of KLRG1 expression in both PBMC and SP (*P* < 0.05) (Figure 3E). Importantly, the number of cells expressing the T cell activation marker, IL-2 receptor α chain CD25, was significantly higher in all three compartments following immunization with NP-C_16_SIL-C_16_R848 (Figure 3F) (Perret et al., 2013; Nishikawa and Sakaguchi, 2014). Together, these results indicate that the co-delivery of adjuvant and peptide antigen augments the development of the antigen-specific CD^8+^ T cell responses, resulting in effector T cells that retain the ability to proliferate and develop into memory CD8^+^ T cells.

### *In vivo* Anti-tumor Immunity of PLN Vaccine

**FIGURE 4:**
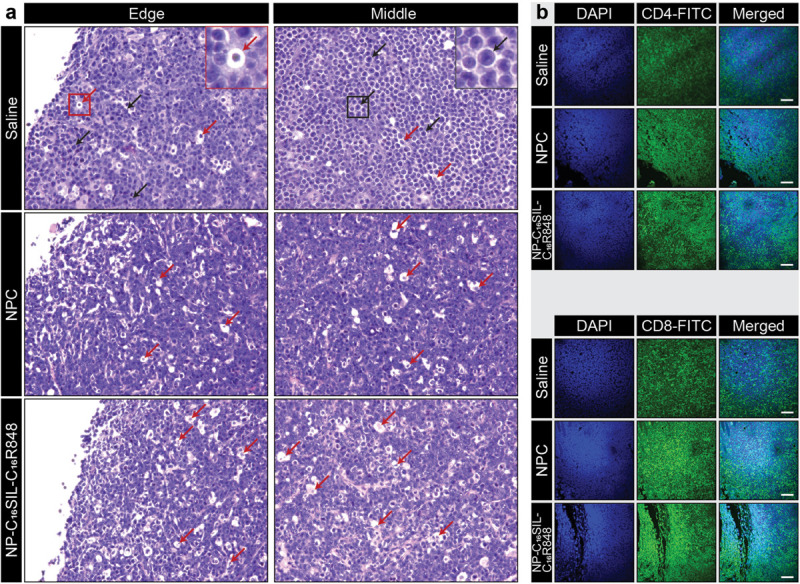
Anti-tumor immunity in the tumor microenvironment (TME). **(a)** Histopathology of representative H&E-stained edge and middle region sections of E.G7-OVA tumors from vaccinated mice. The red arrows indicate apoptotic cells and black arrows indicate normal lymphoma cells. **(b)** Immunofluorescence analysis of CD4^+^ and CD8^+^ T cell infiltration in the tumor tissues in each group. Immunofluorescent staining of nucleus (blue), CD4 or CD8 (green) in tumor slices. Scale bar = 100 μm.

We next sought to determine whether the lipidation approaches could induce desired anticancer immunity against a solid tumor. Tumor growth was significantly reduced in NP-C_16_SIL-C_16_R848 vaccinated groups for more than 2 weeks following inoculation compared with controls (saline, *P* = 0.0005; NPC, *P* = 0.0039) (Figure 3G), leading to improved survival of NP-C_16_SIL-C_16_R848 vaccinated groups of mice (Figure 3H). This prophylactic anticancer efficacy was also observed in the histopathology analysis of the tumor cell morphometry (Figure 4a). Compared with saline and soluble formulations, the NP-C_16_SIL-C_16_R848 immunized group demonstrated a conspicuous increase in the number of apoptotic bodies (red arrow) within the presence of a “starry sky” pattern in both outer edge and inner middle of the tumor lesions. Additionally, the condensation of the chromatin, shrinking of the lymphoma cells, and fragmentation of the nucleus were also more frequently observed. These phenomena are consistent with antitumor immunity through an activation of SIL-specific CD8^+^ response. Furthermore, we explored the distribution of the CD4^+^ and CD8^+^ T cell populations in the tumor tissues of mice and found that CD4^+^ and CD8^+^ T cells labeled by FITC probe are enriched in the tumor nests of both NPC and NP-C_16_SIL-C_16_R848 vaccinated mice, while mice immunized with saline showed less infiltration of CD4^+^ and CD8^+^ T cells in the E.G7 tumor tissue (Figure 4b). These observations demonstrate that our LPN vaccine approach provoked a notable increase of CD4^+^ and CD8^+^ T cells at tumor sites.

## Conclusion

Previously, we showed that co-encapsulation of the model antigen OVA with the TLR7/8 agonist in SVPs improved clinical-grade safety with minimal risk of systemic adverse reactions (Ilyinskii et al., 2014). The present study demonstrates that it is possible to improve anti-tumor immune responses and achieve *in vivo* prophylactic efficacy using a peptide vaccine composed of an SIL epitope with a TLR7/8 agonist, owing to lipidation of adjuvant/peptide epitope and PLN strategy. Our design rationale demonstrated multifaceted benefits, including minimal systemic exposure, enhanced EE, consistent APC uptake, and prolonged immune surveillance. Furthermore, the lipidated PLN formulation approach described here has the potential to be applied beyond TLR-dependent immunostimulatory activity to other adjuvants capable of stimulating innate immune activation. Overall, the combination of medicinal chemistry and formulation science enables the described strategy to address the specific criteria including effective co-encapsulation, *in vivo* stability, prolong release, consistent APC uptake, and safety. Our demonstration could be an important clue to guide the future success of anti-cancer nanovaccines.

## Data Availability Statement

The datasets presented in this study can be found in online repositories. The names of the repositories and accession numbers can be found in the article/Supplementary Material.

## Ethics Statement

The animal study was reviewed and approved by the Brigham and Women’s Hospital Institutional Animal Care and Use Committee.

## Author Contributions

JS and BZ conceived the idea and directed the project. HZ, MI, JW, JR, SD, KL, and YC performed all the experiments and analyzed the data. JW, HZ, and MI wrote the manuscript and revised it according to the comments of JS, JR, and other co-authors. JR and JS provided the technical support and corrections of the manuscript. All authors contributed to the article and approved the submitted version.

## Conflict of Interest

JR was employed by the company Silicon Therapeutics, Boston, MA, United States. MI was employed by the company Immuno-Oncology Group, Immunomic Therapeutics, Inc., Rockville, MD, United States.

The remaining authors declare that the research was conducted in the absence of any commercial or financial relationships that could be construed as a potential conflict of interest.
