# Supplementary material for: Lipidation Approaches Potentiate Adjuvant-Pulsed Immune Surveillance: A Design Rationale for Cancer Nanovaccine
Source: Front Bioeng Biotechnol. 2020 Jul 28;8:787. doi: 10.3389/fbioe.2020.00787 (PMC7399020; doi:10.3389/fbioe.2020.00787)
Supplement: Supplementary file 1 [file Data_Sheet_1.docx]

Lipidation Approaches Potentiate Adjuvant-pulsed Immune Surveillance: A Design Rationale for Cancer Nanovaccine

Junqing Wang^1,4†^, Harshal Zope^1†^, Mohammad Ariful Islam^1,5†^, Jamie Rice^2,3^, Sage Dodman ^1^, Kevin Lipert^1^, Yunhan Chen^1^, Bruce R. Zetter^2^, Jinjun Shi^1^*

^1^Center for Nanomedicine and Department of Anesthesiology, Brigham and Women’s Hospital Harvard Medical School, Boston, MA, United States

^2^Vascular Biology Program, Boston Children’s Hospital, Harvard Medical School, Boston, MA, United States

^3^Silicon Therapeutics Boston, MA, United States

^4^School of Pharmaceutical Sciences (Shenzhen), Sun Yat-sen University, Guangzhou, China.

^5^Immuno-Oncology Group, Immunomic Therapeutics, Inc., Rockville, Maryland, USA

*** Correspondence:**Jinjun Shi
[jshi@bwh.harvard.edu](mailto:jshi@bwh.harvard.edu)

^†^These authors have contributed equally to this work

**Table of Contents**

[In Vivo Experiments (Tumor Inoculation and Measurement) 1](#_Toc39996676)

[Figure S1 4](#_Toc39996677)

[Figure S2 5](#_Toc39996678)

[Figure S3 6](#_Toc39996679)

[Figure S4 7](#_Toc39996680)

[Figure S5 8](#_Toc39996681)

[Figure S6 8](#_Toc39996682)

[Figure S7 9](#_Toc39996683)

[Figure S8 10](#_Toc39996684)

## In Vivo Experiments (Tumor Inoculation and Measurement)

Tumors were inoculated four days after the last vaccination round. EG7-OVA cells were suspended in media at the concentration of 1×105 cells per 100 uL. 100 uL of Matrigel was added for each 100 uL of cell solution. Each mouse was inoculated subcutaneously on the back with 200 uL of the above mixture. Mice were checked daily for tumor growth. Noticeable tumors were observed 10 days after initial implantation and the first measurement were taken. Each group was measured daily. Mice were sacrificed when the overall volume of the tumor (calculated by simple multiplication of length, width, and height) reached 2000 cubic mm. All mice from all groups were sacrificed by day 19.

Blood Draw. Approximately 200 uL of blood was collected via retro-orbital or submandibular bleed (using a heparinized capillary tube or lancet respectively) into the correctly labeled heparinized tube. The collected blood samples were stored on ice.

Preparation for FACS. After the blood draw, 600 uL of histopaque (HP1083) was carefully added to each glass tube. The glass tube was then placed into a 15 mL falcon tube (Corning). Falcon tubes were then centrifuged for 35 minutes, 350g. A mix of 5 uL of SIINFEKL-H2K^b^ PE tetramer solution in 90 uL of FACS buffer was made for each sample. Also, make a mix of 3 uL each of CD8a FITC, CD127 BV785, CD44 BV570, KLRG1 PerCP-Cy5.5 and CD25a BV42 per sample and 2 uL each of CD4 BV650, CD19 BV650, and CD11b BV650 per sample. This mixture was protected from light and stored in 4^o^C refrigerator until needed. 3 mL of cold 1X PBS was added to each of 23 FACS tubes. The 15 mL falcon tubes were removed and placed into a rack. The cloudy layer between the histopaque and the blood was removed and transferred into a corresponding FACS tube with cold PBS. The FACS tubes with the cell layer were placed into an icebox immediately after the collection. The tubes with the cell layers were then spun at 350g and 4^o^C for 5 minutes. The supernatant was aspirated. The cells were then resuspended in 1 mL of cold PBS and counted. In each tube, a minimum of 1.5 million cells was preferred. After all samples were counted, a total of 1 million cells from each of the tubes containing the most cells were removed. These were added into new FACS tubes. They were resuspended in 2 mL of 1X PBS and placed on ice for later use. The remaining cell samples were each resuspended in 95 uL of the FACS/tetramer solution and incubated at room temperature in the dark (placed in a drawer) for 20 minutes. After incubation, 21 uL of the antibody cocktail from above was added to each sample and incubated again for 20 minutes in the dark at room temperature. During this incubation, the flow cytometer (EMD Millipore) was turned on and cleaned if necessary. Once the incubation was complete, 2mL of FACS buffer was added to each tube and they were spun at 350g and 4^o^C for 5 minutes, after which the supernatant was aspirated. 3 mL of FACS buffer was added to each tube and the wash process was repeated. Each sample was resuspended in 100 uL of FACS buffer. The FACS tubes containing the extra cells were gently pipetted. The suspensions were distributed evenly between 8 FACS tubes. 2 mL of cold PBS was added to each FACS tube, and were centrifuged at 350g for 5 minutes at 4^o^C. After the supernatant was aspirated, the cells in each tube were resuspended in 100 uL of FACS buffer. Antibody was added to each tube as follows: 3 uL CD8a FITC, 5 uL CD25 PE only, 3 uL KLRG1 PerCP-Cy5.5, 3 uL CD127 BV785, 3 uL CD44 BV570, 2 uL CD4 BV650, and 3 uL CD25a BV421. Samples were incubated for 15 minutes at room temperature in the dark. 2 mL of PBS was added. Each was resuspended and aspirated in 150 uL of FACS buffer after centrifugation. The FACS settings and analysis protocols were the same as those used at the end of the in vivo section.

Sacrifice of Mice and Processing of Organs. Mice to be sacrificed were first anesthetized with isoflurane. Then, blood was drawn via the retro-orbital method in which the procedure was the same as the blood draw method detailed above. Mice were then sacrificed using the cervical dislocation method. First, four lymph nodes were removed – two auxiliary and two inguinal. Then, the spleen was removed. Each removed organ was placed in a well of a 24-well plate filled with sample preparation media, which consisted of DMEM, FBS, HEPES, β-mercaptoethanol, and penicillin-streptomycin. The tumor was removed from the dorsal side and placed in a well in a 6 well-plate filled with cold 1X PBS. The plates were then stored at 4°C for later processing.

After photographic documentation of tumors, each was split in half. One half of the tumor was cut into quarter to eighth-sized pieces and dropped into 4% PFA solution in labeled plastic bottles for fixing. These were then transferred into 70% ethanol after 2 days for long-term storage. The other half was sectioned into small horizontal and vertical slices, then mounted in optimal cutting temperature (OCT) compound deposited in a labeled plastic holder. These holders were carefully arranged on a Styrofoam board, covered with aluminum foil, and placed in -80°C to allow the OCT compound to freeze. 70 uM strainers (Corning) were placed in fitting 50 mL falcon tubes (Corning). They were wetted with cold sample prep media. Each spleen or set of four lymph nodes was placed in a strainer and mashed with the sterile plunger bottom of a 5 mL syringe. The mash was then rinsed with more cold media. The collected cell media solution on the bottom of the tube was transferred – spleen solutions were transferred to 15 mL falcon tubes (Corning) and lymph node solutions to FACS tubes. 40 uL of MACS buffer (consisting of 1X PBS, BSA, and EDTA) was then added to the solution along with 10 uL of MACS biotin-antibody cocktail. Both splenocytes and lymph node cells were counted. By throwing out excess lymph node cells and transferring the appropriate number of splenocytes to new FACS tubes, 1.5 million cells were kept from each cell sample. For each cell sample, 3 mL of FACS buffer was added for resuspension. The samples were spun down and aspirated. A SIINFEKL-H2K^b^ PE tetramer solution was made by mixing 5 uL of that tetramer with 90 uL of FACS buffer per sample. A labeling cocktail for was made by mixing assorted antibodies (consisting of the following – CD8a FITC, CD127 BV785, CD44 BV570, KLR1 PerCP-Cy5.5, CD25a BV 421, and CD62L PE-Cy7 – 3 uL of each per sample; CD4 BV650, CD19 BV650, and CD11b BV650 – 2 uL of each per sample). Each cell sample was first resuspended in 95 uL of tetramer solution and incubated at room temperature in the dark for 20 minutes. 24 uL of the antibody mix was subsequently added per sample, which were incubated for 20 additional minutes at room temperature. 2 mL of FACS buffer was added to each sample, which was then spun down and aspirated. This wash was then repeated one more time. Each sample was finally resuspended in 250 uL of FACS buffer that has 0.2% PFA. They were then transferred to a round-bottom, clear 96-well plate for FACS analysis. To make experiment standards for FACS, 1 drop of negative control beads were added to each FACS tube, making 9 tubes total. Then, 1 uL of a selected antibody was added to each tube except for one, resulting in the following controls – unlabeled, CD8 FITC only, CD25 PE only, KLRG1 PerCP-Cy5.5 only, CD127 BV785, CD44 BV570, CD4 BV650, CD25a BV421, and CD62L PE-Cy7. This mixture was incubated for 10 minutes at room temperature. 2 mL of FACS buffer was added, and the mixture was spun down and aspirated. Finally, the labeled beads were resuspended in 150 uL of FACS buffer, which were then transferred to the plate above.


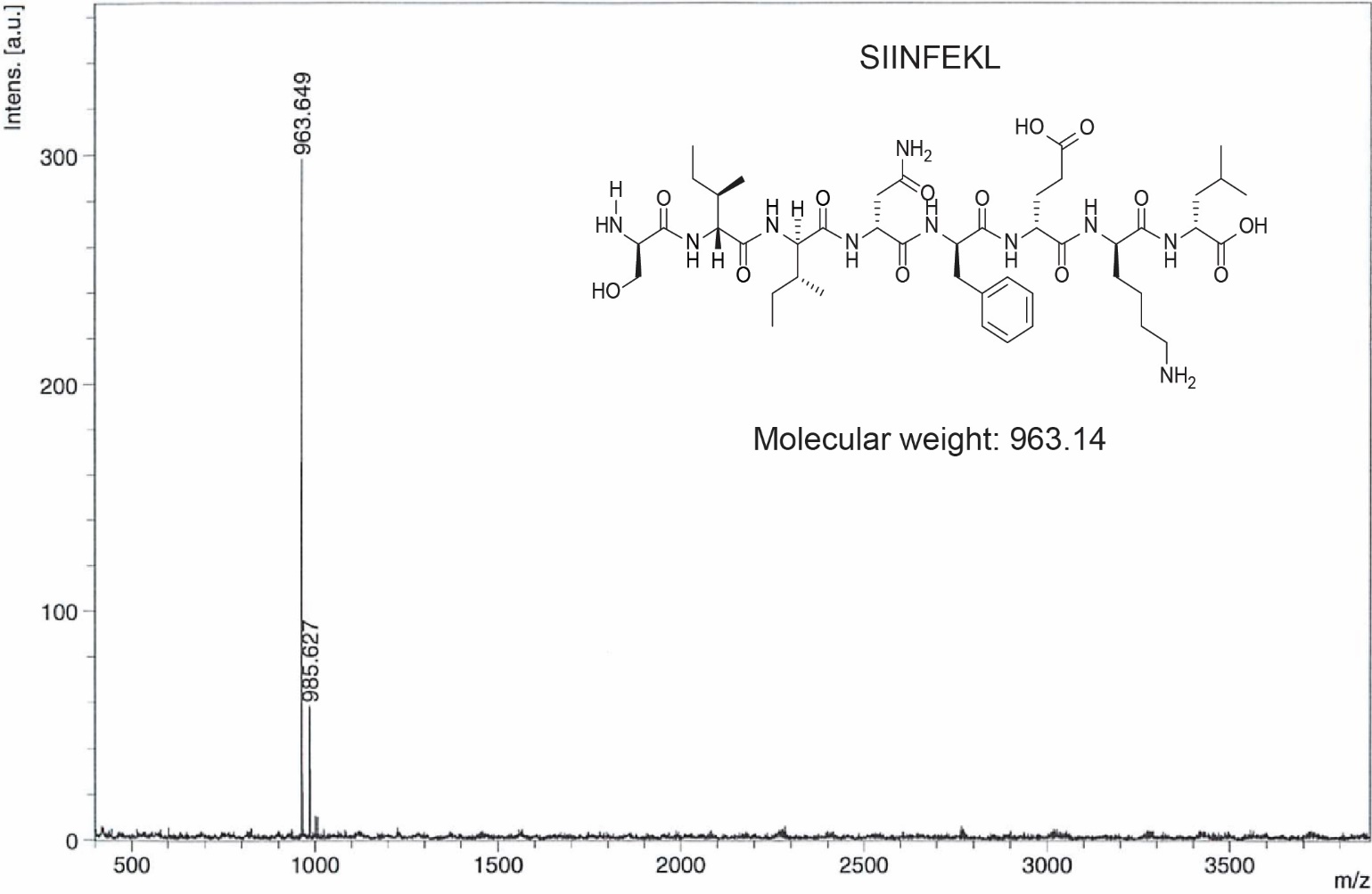


Figure S1***.*** The Mass spectrometry identification of OVA257-264 (SIINFEKL) peptide.


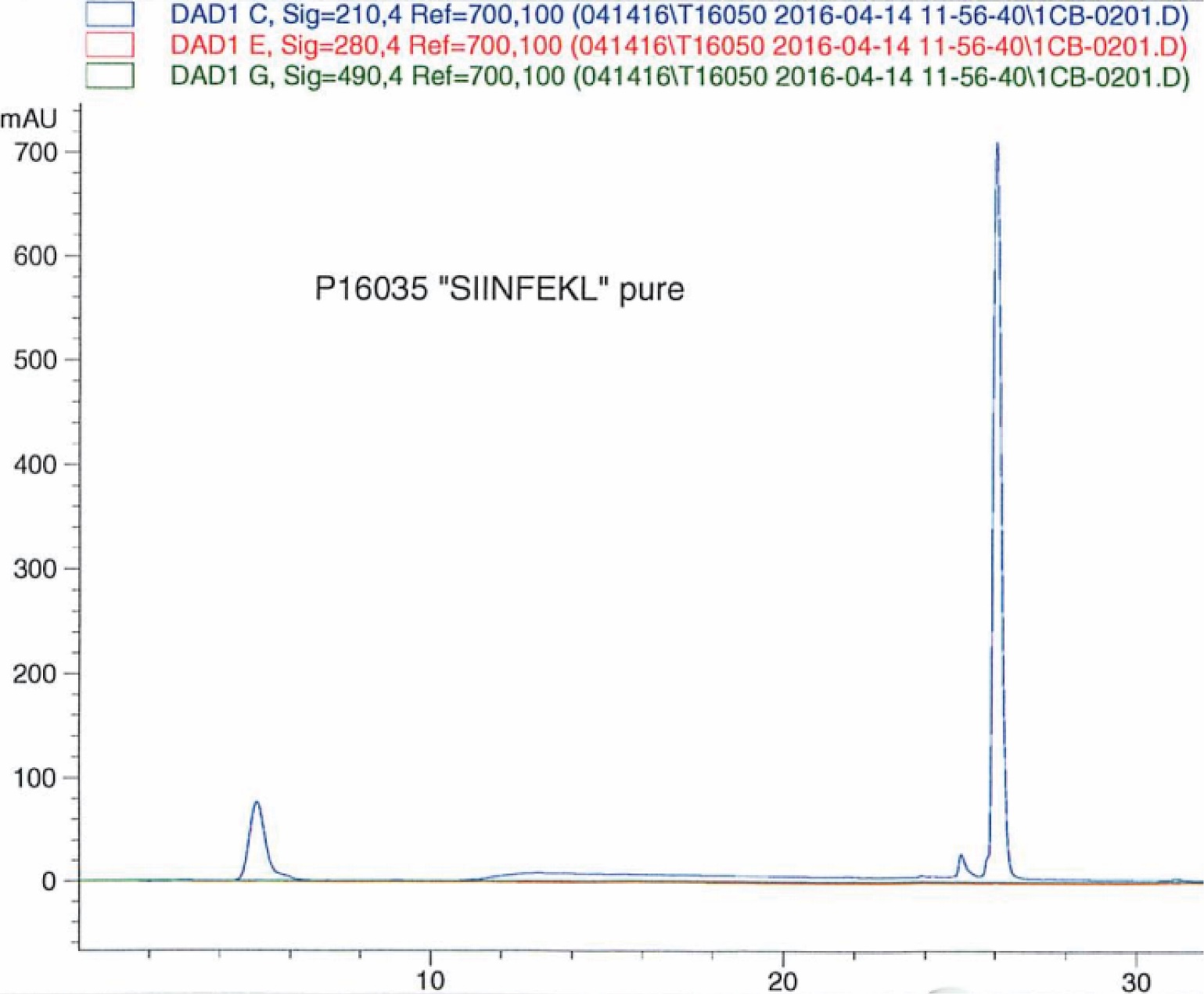


Figure S2***.*** The Chromatogram Graph of OVA257-264 (SIINFEKL) peptide.


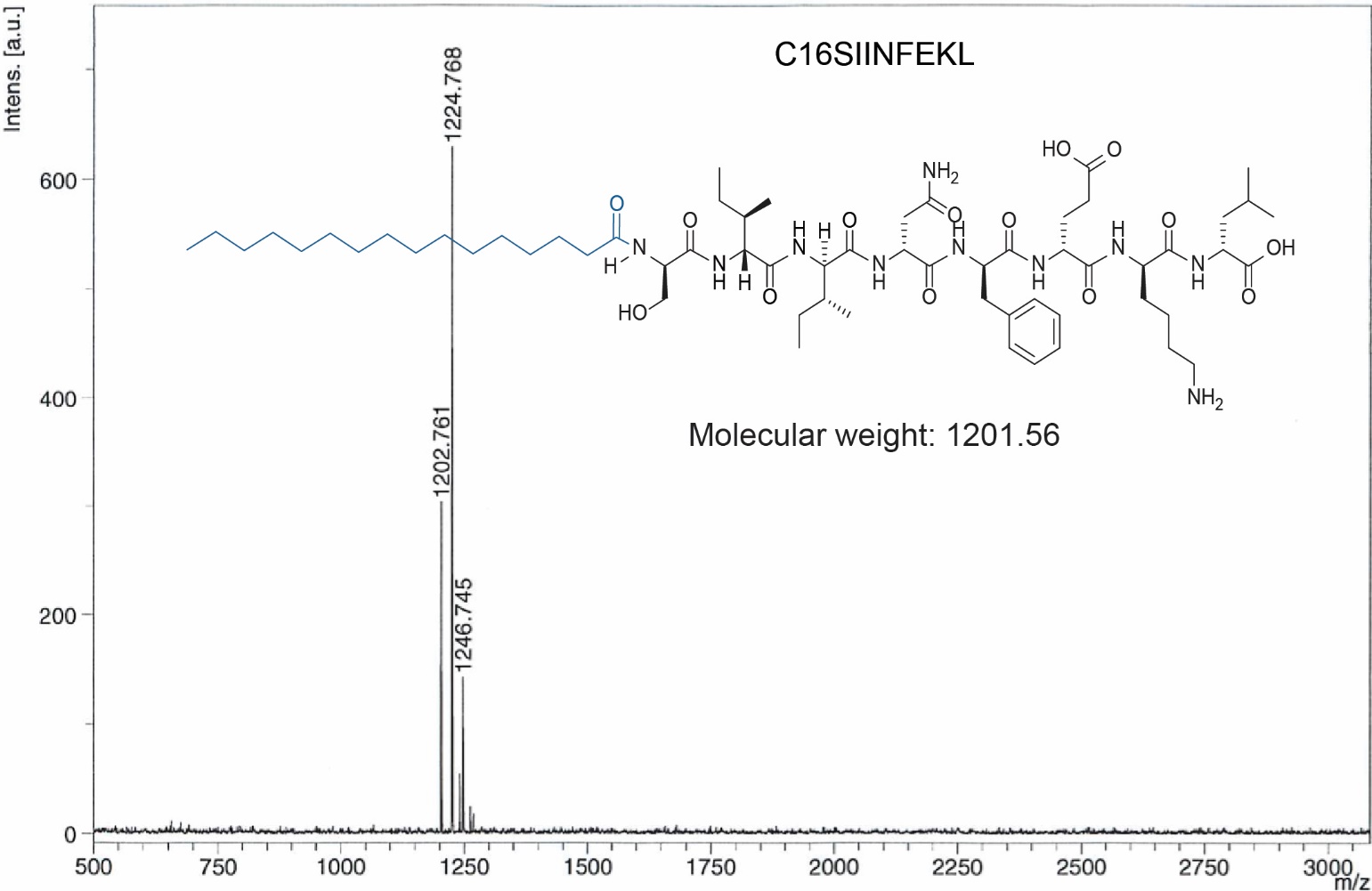


Figure S3***.*** The Mass spectrometry identification of obtained compounds C_16_SIINFEKL.


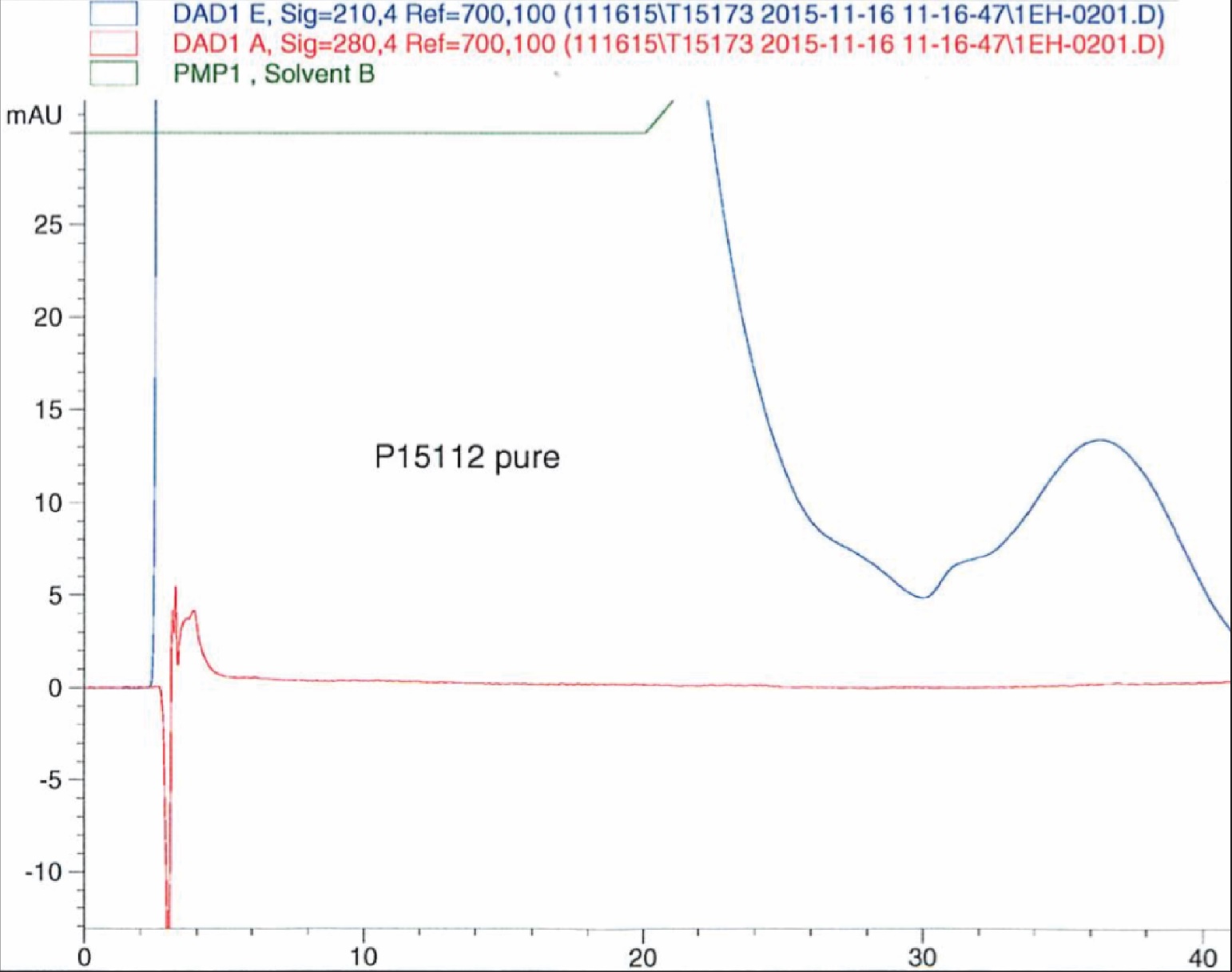


Figure S4***.*** The Chromatogram Graph of obtained compounds C_16_SIINFEKL.


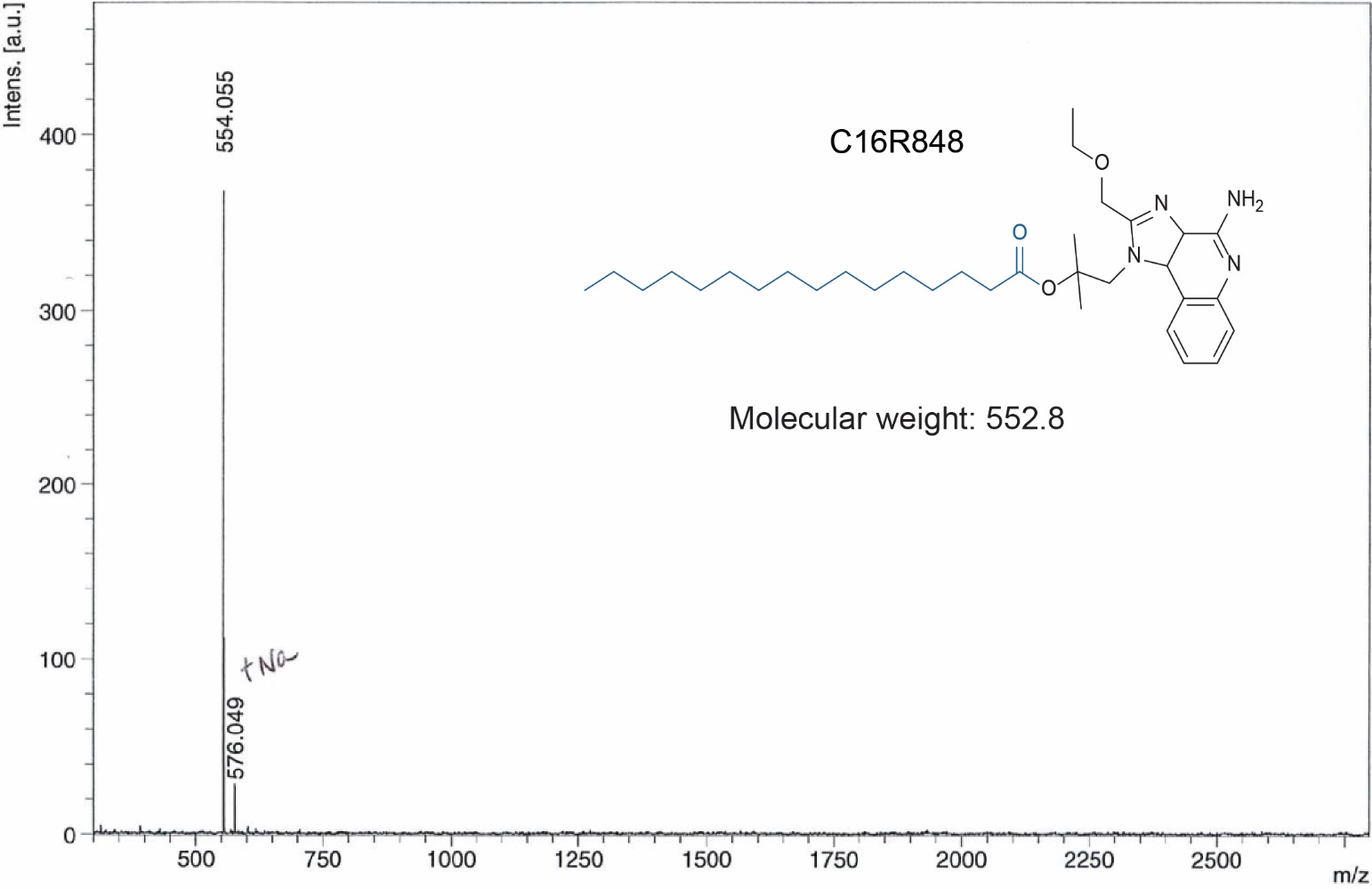


Figure S5***.*** The Mass spectrometry identification of obtained compounds C_16_R848.


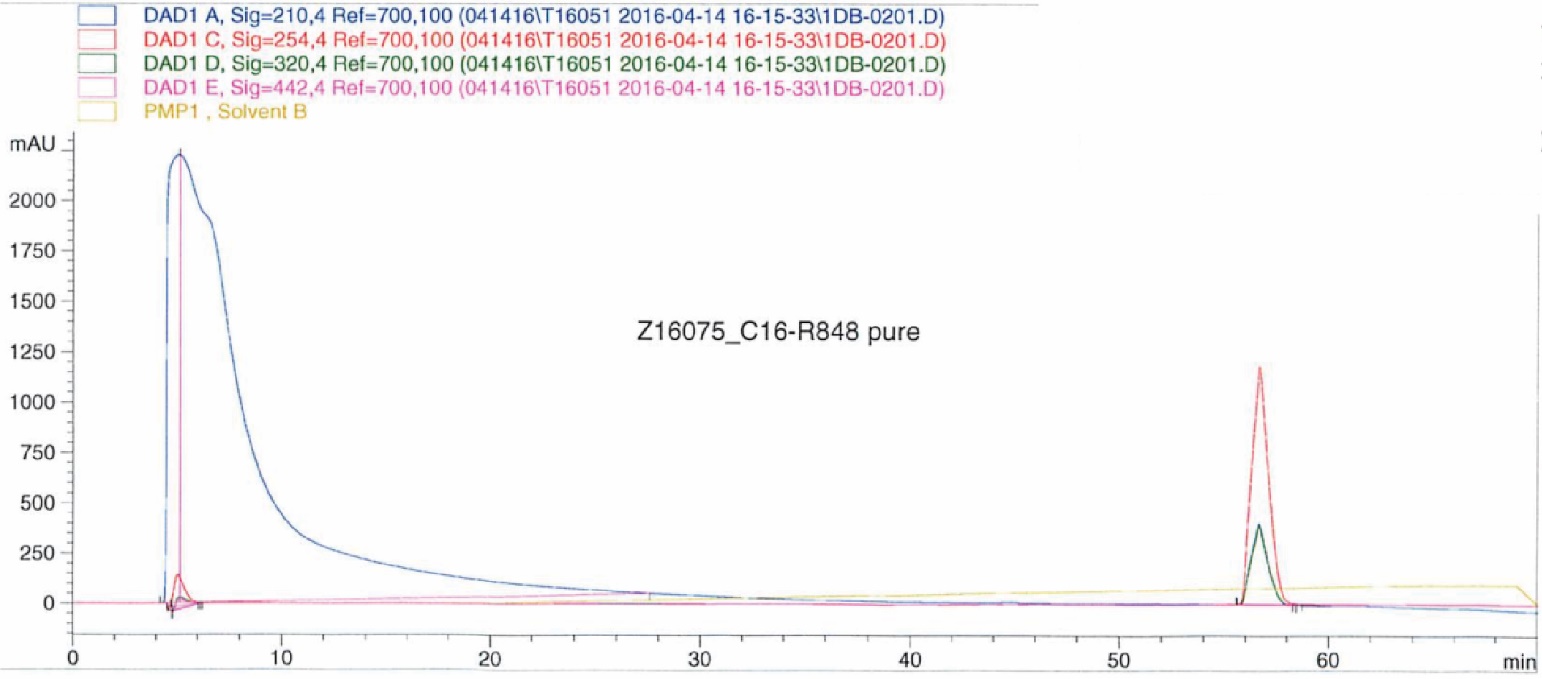


Figure S6***.*** The Chromatogram Graph of obtained compounds C_16_R848.


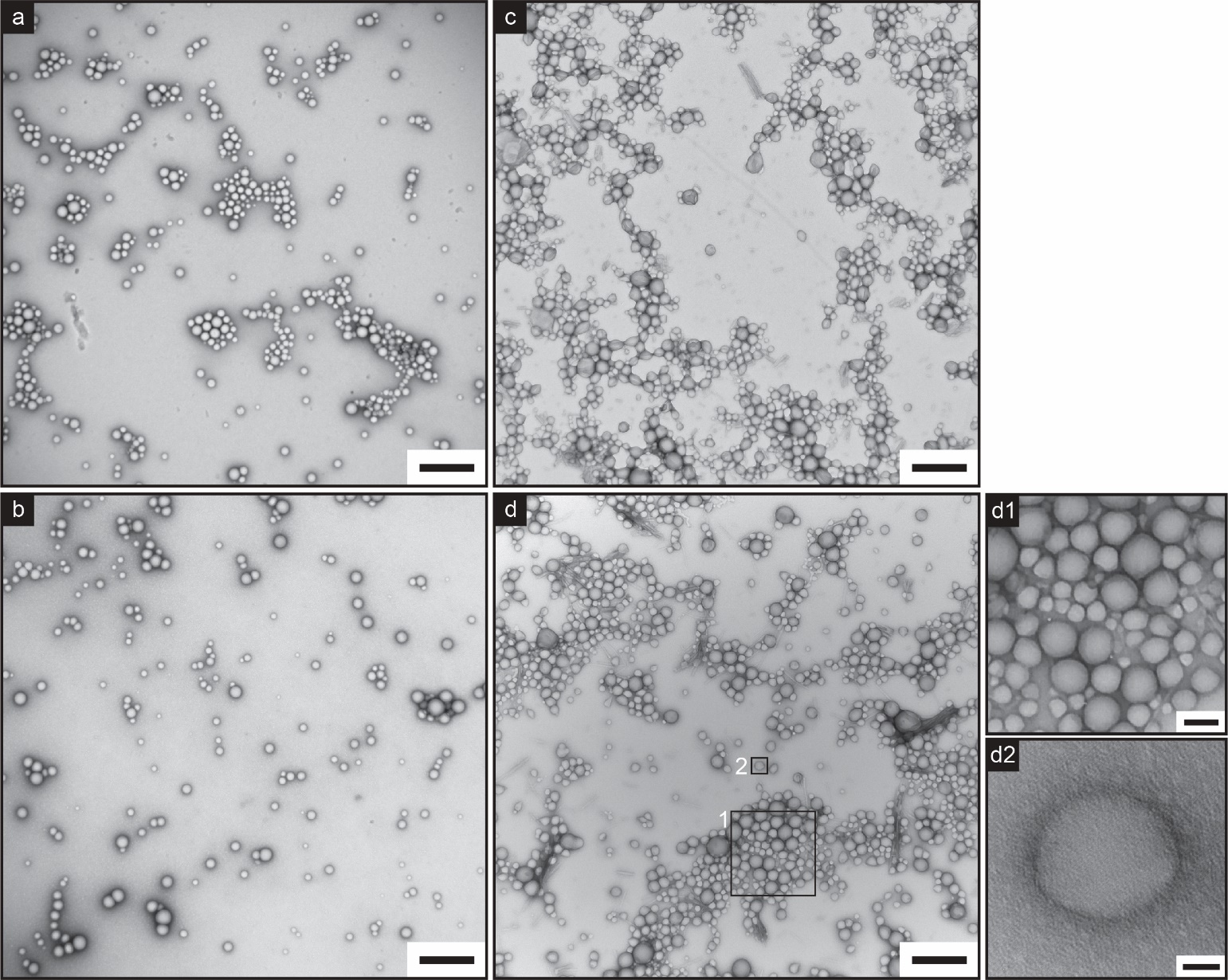


Figure S7***.*** The representative TEM images of prepared PLNs: a) PLA–CerPEG (empty-NP), b) PLA–C_16_R848–CerPEG (NP-C_16_R848), c) PLA–C_16_SIL–CerPEG (NP-C_16_SIL), d) PLA–C_16_SIL/–C_16_R848–CerPEG (NP-C_16_SIL-C_16_R848).


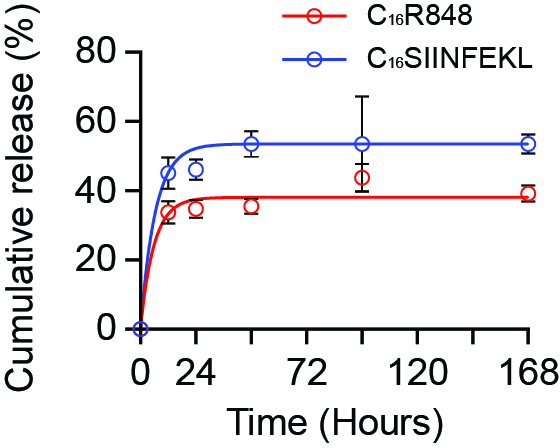


**Figure S8.** *In vitro* drug release profile of PLN (C_16_R848 and C_16_SIL) up to 168 hours.


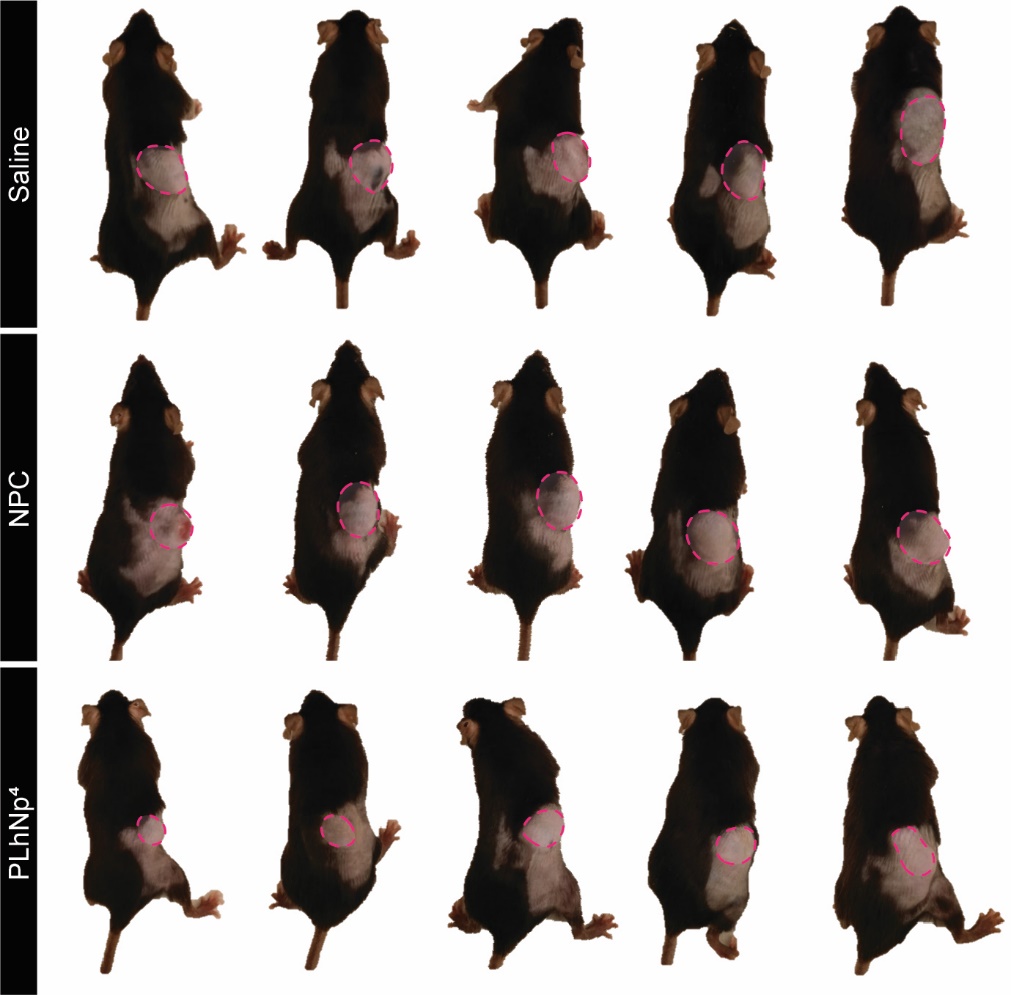


Figure S9*.* Images showing the E.G7-OVA thymic lymphoma xenografted C57BL/6 mice in three treatment groups. The upper panel indicates mice treated with saline solution, the middle panel indicates mice treated with NPC formulation and the lower panel indicates mice treated with NP-C_16_SIL-C_16_R848 formulation.


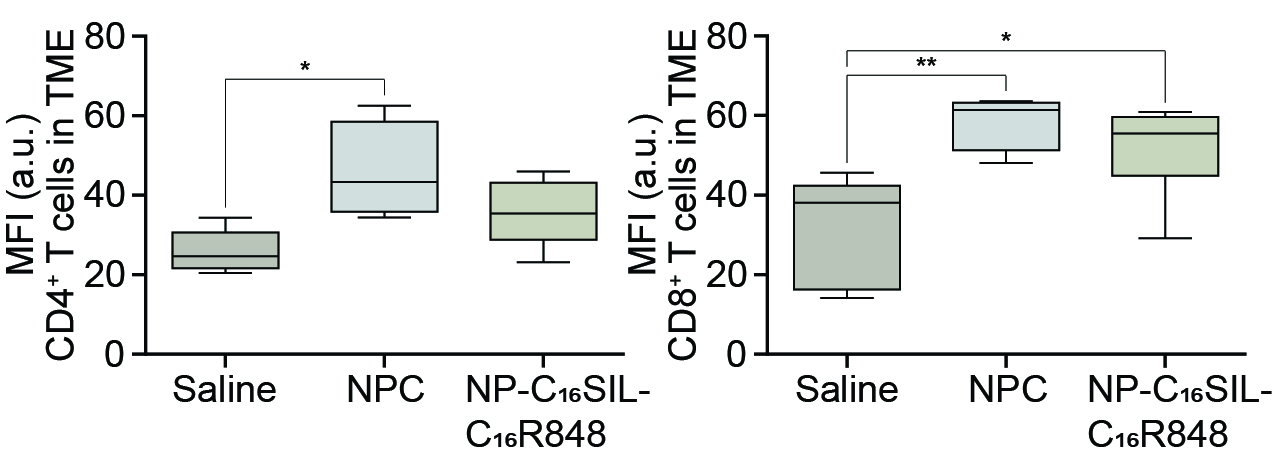


**Figure S10***.* Quantitative mean fluorescence intensity (MFI) analysis of the percent area of the tumor slices. *P < 0.05, **P < 0.01 analyzed by one-way ANOVA.
